# Socioeconomic inequalities in type 2 diabetes mellitus: a study based on a population-based survey in Iran

**DOI:** 10.1186/s12889-024-18452-7

**Published:** 2024-03-30

**Authors:** Ali Darvishi, Adeleh Nikkhah, Marzieh Mahmudimanesh, Narges Zargar Balajam, Gita Shafiee, Ramin Heshmat

**Affiliations:** 1 Chronic Diseases Research Center, Endocrinology and Metabolism Population Sciences Institute, Tehran University of Medical Sciences, Tehran, Iran; 2 Endocrinology and Metabolism Research Center, Endocrinology and Metabolism Clinical Sciences Institute, Tehran University of Medical Sciences, Tehran, Iran; 3 Endocrinology and Metabolism Research Institute, No. 10, Jalale- Al-Ahmad Ave, Chamran Highway, Tehran, Iran

**Keywords:** Socioeconomic inequality, Type 2 diabetes, Concentration index, Iran DiaCare

## Abstract

**Background:**

Type 2 diabetes mellitus (T2DM) is the most prevalent form of Diabetes Mellitus (DM), with social and economic determinants significantly influencing its prevalence. This study aimed to analyze the socioeconomic inequalities associated with T2DM in Iran.

**Methods:**

Data from an observational survey in Iran, titled “Diabetes Care (DiaCare),” were utilized for this study. Socioeconomic inequalities were assessed through variables including Hemoglobin A1C (HbA1c), Fasting Blood Glucose (FBG), and Triple target (HbA1c, blood pressure, LDL-C), using concentration indices (CIs) and a multivariate logistic regression analysis. Individual socioeconomic status (SES) was determined by calculating an asset index using principle component analysis (PCA) based on their properties. Data analysis was conducted using STATA software version 14.

**Results:**

A total of 13,321 participants were included in the study. The CIs were significantly positive for controlled HbA1c (0.0324) and triple target (0.1067), while for controlled FBG, it was 0.0125, although not significant. Among females, the CIs were significantly positive for controlled HbA1c (0.0745), FBG (0.0367), and triple target (0.209). Additionally, in the 45–55 and 65–75 age groups, the CIs were significantly positive for controlled HbA1c (0.0607) and FBG (0.0708), respectively. This index was significant for controlled Triple target in the 35–45 (0.376) and 65–75 (0.124) age groups. The CI for controlled FBG was significant in rural dwellers (-0.044) while the concentration of controlled triple target was significant in urban dwellers (0.0967). Controlled HbA1c showed significant concentration in both urban (0.0306) and rural (-0.0576) dwellers. Furthermore, the CIs were significant for controlled HbA1c in regions with medium prevalence (0.0534) and FBG in regions with low prevalence (-0.0277). This index was significantly positive for controlled triple target in regions with high prevalence (0.124).

**Conclusions:**

Diabetes care is more concentrated among individuals with higher SES. Policymakers should consider this to mitigate the inequality and alleviate the burden of T2DM.

## Background

Diabetes Mellitus (DM), as a chronic non-communicable disease (NCD), is one of the most important public health problems that affects negatively the lives of millions worldwide [1]. It serves as a primary contributor to both mortality and morbidity, resulting in escalated healthcare costs [2, 3]. Due to the silent nature of diabetes, a significant portion of the affected population is unaware of their condition [4]. According to the International Diabetes Federation (IDF) report, there is a projected increase in diabetes cases from 88 million in 2019 to 153 million in 2045 [5]. Furthermore, healthcare expenditures associated with diabetes in 2021 were estimated at approximately $966 billion, with projections indicating an increase to $1028 billion by 2030 [2].

Evidence demonstrates that diabetes can lead to heart disease, damage to the eyes, kidneys, and nerves, an elevated risk of amputation, disability, and ultimately death [2, 6].

In general, the primary types of diabetes include type 1, type 2, and gestational diabetes. Type 2 diabetes mellitus (T2DM) is more prevalent and occurs when the body produces insulin but is unable to use it effectively [3, 7]. Individuals with T2DM may remain unaware of their condition for an extended period as the symptoms can take many years to manifest. Throughout this duration, elevated blood glucose levels can lead to destructive damage within the body [3]. The management of diabetes involves various factors, including medication, lifestyle modifications such as physical activity, and a balanced diet [3]. Controlling blood sugar is a preventive measure to mitigate vascular complications related to diabetes. Effective glycemic control is indicated by a Glycosylated Hemoglobin (HbA1c) level of less than or equal to 6.5% and a Fasting Blood Glucose (FBG) level of less than 7 mmol/L [3].

Studies indicate a continuous rise in the prevalence of T2DM in recent years, attributed to factors such as genetics, population growth, aging, urbanization, obesity, inactivity, and unhealthy diet [7–9]. Beyond genetic and environmental influences, social and economic determinants also contribute to T2DM prevalence. Socioeconomic status (SES) encompasses factors such as access to healthcare and information, availability of healthy foods and sports facilities, income level, education, job opportunities, and individual lifestyle choices [1, 3, 8].

Despite the widespread coverage of healthcare systems, certain countries exhibit socioeconomic inequalities [10]. Socioeconomic inequalities are characterized by an uneven distribution of access to resources and opportunities, hindering the achievement of a healthy lifestyle [8]. Access to health care, treatment choices, and control recommendations for the treatment of T2DM are influenced by socioeconomic factors [11].

A study (2022) emphasized the association between socioeconomic inequalities and the rise in the prevalence of T2DM [8]. In another study, socioeconomic inequalities were reported based on the gender and education level of patients with T2DM [12].

Given the significant burden of diabetes in Iran, the health system has implemented various programs to alleviate the effects and burden of this disease in the country. These initiatives include the National Diabetes Prevention and Control Program and the National Action Plan for the Prevention and Control of NCDs, among others. It has been observed that in all these programs and policies, reducing inequality at different levels has consistently been one of their defining goals [13–15]. However, the extent to which these programs have been successful and have achieved their goals needs to be carefully examined.

Understanding socioeconomic inequalities is crucial for achieving justice in healthcare. Having information about factors related to these inequalities, especially among patients with T2DM can lead to the design of programs to reduce the unequal distribution of healthcare in the future. Therefore, this study was conducted to analyze the socioeconomic inequalities of T2DM related variables in Iran.

## Methods

The present cross-sectional study utilized data from an observational, population-based survey entitled “Diabetes Care (DiaCare)” [16]. DiaCare was a nationwide study conducted between 2018 and 2020, aimed at assessing the care status of individuals with T2DM across all thirty-one provinces of Iran. Participants, aged 35–75 years and residing in both urban and rural areas, were surveyed to form a representative sample. DiaCare employed various techniques to select primary sampling units, including systematic random sampling, stratified sampling, and cluster sampling. The sample size was consistent across provinces, with the division between urban and rural areas based on their population proportions within the provinces [16].

The inclusion criteria involved a diagnosis of T2DM following the recommendations of the American Diabetes Association (ADA) [17]. Data collection was carried out through interviews using a questionnaire. Various variables were gathered, including SES, fasting blood glucose (FBG), HbA1c, and Triple target (HbA1c, blood pressure, LDL-C). Additional details about the DiaCare study can be referenced in the published study protocol [16].

In this study, socioeconomic inequalities were calculated to investigate T2DM-related variables including controlled HbA1c, FBG, and the Triple target.

Information about SES of individuals was assessed based on assets, including house ownership status, house area, house chamber, and possession of items such as TV, LED/LCD TV, landline, mobile phone, refrigerator, washing machine, dishwasher, microwave, laptop, internet access, and car. Principle Component Analysis (PCA) was employed to compute the SES index (asset index) of individuals based on their properties. Subsequently, the calculated asset index was divided into five quintiles. The first quintile included the poorest individuals, while the fifth quintile consisted of the richest.

Socioeconomic inequality is measured by the Concentration Index (CI), which ranges from − 1 to + 1. When CI is positive (negative), the desired variable is distributed among individuals with high (low) SES, while a value of zero indicates equality. The CI, along with the associated concentration curve, measures socioeconomic-related inequality in a particular health variable. The CI is twice the area between the concentration curve and the equity diagonal line (the 45-degree line). If desired variable is distributed equally across every quintile of the population (CI = 0), the concentration curve be located on equity diagonal line. The concentration curve would be placed above the diagonal line (CI is negative) if health is concentrated among poor levels of the people. Finally, if the concentration curve would be located under the diagonal line (CI is positive), desired variable is concentrated among rich people.

In present study along with considering controlled HbA1c, FBG, and the Triple target variables in whole sample, CI was also measured across different demographic groups. This includes sex groups (male and female), age groups (35–45, 45–55, 55–65 and 65–75 years old) and also by place of residence (rural and urban). Finally, based on the prevalence of T2DM, Iran’s provinces were divided into three categories: low, medium, and high prevalence. Subsequently, socioeconomic inequalities of mentioned variables were assessed based on these categories.

To elucidate the impact of socioeconomic status (SES) on diabetes management outcomes, we conducted a multivariate logistic regression analysis, delineating the association between the quintiles of the asset index and the achievement of controlled Triple target, FBG, and HbA1c levels. The analysis was structured in a stepwise manner: a crude model considering only the asset index quintiles; Model 1, which adjusted for age and sex; and Model 2, which further adjusted for education. This stratified approach allowed for a nuanced understanding of the influence of socioeconomic factors on diabetes control, accounting for potential confounding variables. The logistic regression models aimed to provide odds ratios (ORs) with 95% confidence intervals (CIs), facilitating a robust analysis of the data. Statistical significance was evaluated at *p* < 0.05.

Data analysis was conducted using STATA software version 14.

## Results

In total, 13,321 patients with T2DM were included in the study, among whom 50.17% were women. The percentage and distribution frequency of assets are presented in Table 1. Most individuals had house ownership (80.9%), landline (81.6%), mobile phone (94.4%), and washing machine (85.7%).

Table 2 shows frequency distribution of patients by asset categories. Among the richest group, the patients with T2DM were younger (52.65 ± 8.62) and had a higher level of education than other quintiles. Also, they were mostly urban dwellers (94.5%).

**Fig. 1:**
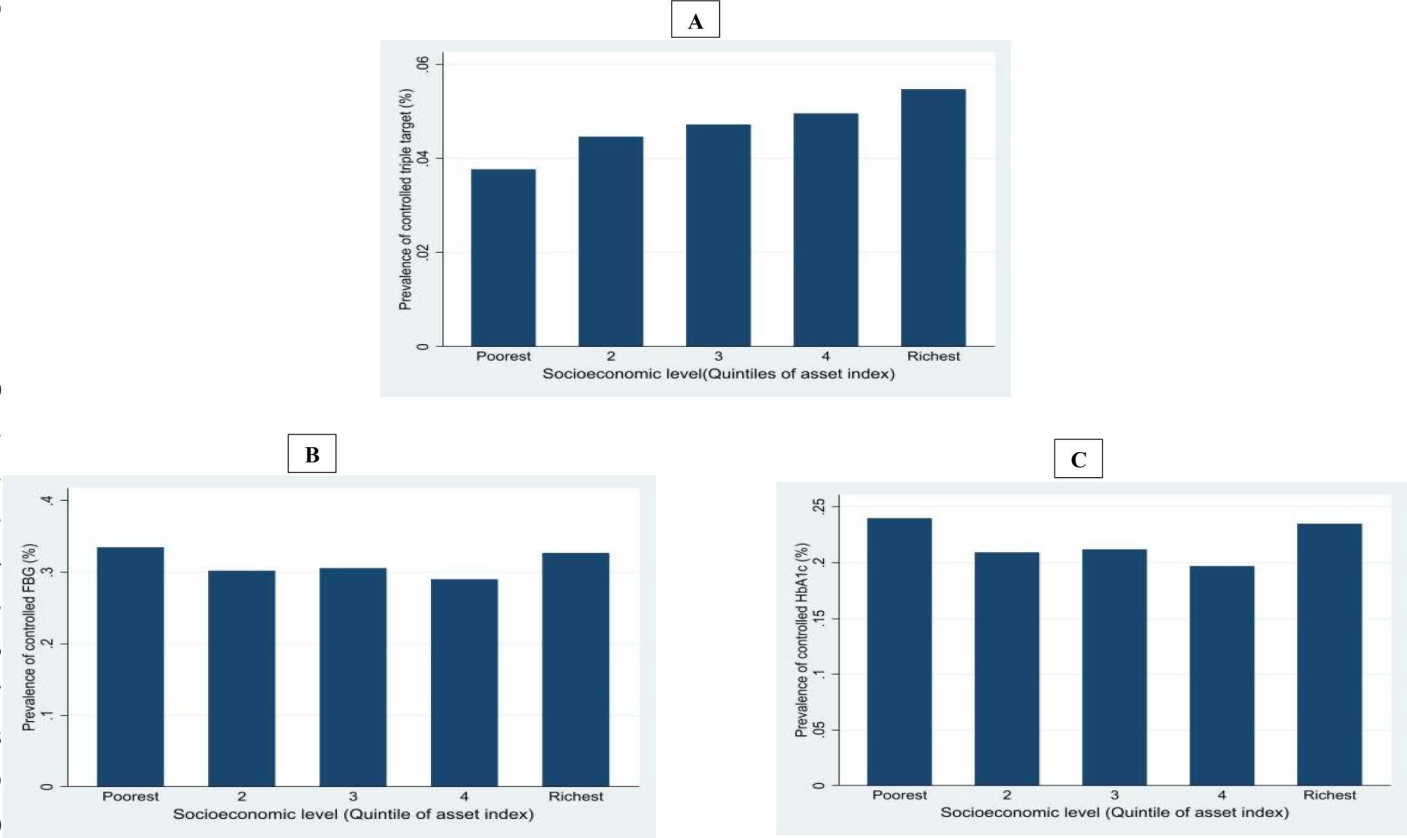
Prevalence of controlled triple target (**A**), controlled FBG (**B**), and controlled HbA1c (**C**) in different socioeconomic status

Figure 1 illustrates the prevalence of diabetic control in asset index quintiles. The prevalence of the controlled triple target was significantly higher among the richest group. However, the prevalence of controlled FBG and HbA1c was higher among the poorest group.

**Table 1:** Frequency distributions of assets

Assets	Frequency (%)	Assets	Frequency (%)
House Ownership	11,654 (80.9)	Refrigerator	7643(60.6)
House Area > 100 m^2^	6967(35.8)	Washing machine	10,262(85.7)
House Chamber ≥ 3	4799(24.8)	Dishwasher	1305(15.4)
TV	5917(38.0)	Microwave	2464(24.2)
LED/LCD TV	8592(73.6)	Laptop	3172(26.3)
Landline	9564(81.6)	Internet access	4111(38.2)
Mobile phone	12,346(94.4)	Car	6743(52.8)

Data as presented as number(percent)

**Table 2:** Frequency distribution of patients with T2DM based on asset categories

Variables	Quintile 1	Quintile 2	Quintile 3	Quintile 4	Quintile 5	P-value
**Sex**						0.003
Men	1072 (40.9)	1246 (46.2)	1297 (46.5)	1410 (52.4)	1613 (57.3)	
Women	1599 (59.1)	1411 (53.8)	1367 (53.5)	1255 (47.6)	1049 (42.7)	
Age (Years)	56.85 ± 9.93	55.70 ± 9.46	54.98 ± 9.42	54.15 ± 9.22	52.65 ± 8.62	< 0.001
**Place of residence**						< 0.001
Urban	1294 (57.7)	1733 (75.6)	1943 (84.5)	2166 (89.6)	2387 (94.5)	
Rural	1377 (42.3)	924 (24.4)	721 (15.5)	499 (10.5)	275(5.5)	
Education (Level)	0 (0–5)	4 (0–6)	5 (1–9)	6 (4–12)	12 (6–14)	< 0.001
Duration of T2DM diagnosis (Years)	60 (36–120)	72 (36–124)	72 (36–120)	72 (36–125)	72(36–120)	0.1

Data are presented as number(percent), mean ± standard deviation (SD), and median (interquartile range)

The findings of the CI for T2DM variables have been presented in Table 3, categorized by sex groups, age groups, place of residence, and prevalence of T2DM. The CI for the controlled FBG variable in the entire population was positive and very close to zero, but it was not statistically significant. There was no significant difference observed between the socioeconomic groups in terms of this variable. In contrast, the CI for the controlled triple target variable was 0.1067 and highly significant (< 0.001), indicating a greater concentration among people with higher SES.

Also, the CI for the controlled HbA1c was 0.0324 and statistically significant (0.0001), indicating it concentrates among people with higher SES.

**Fig. 2:**
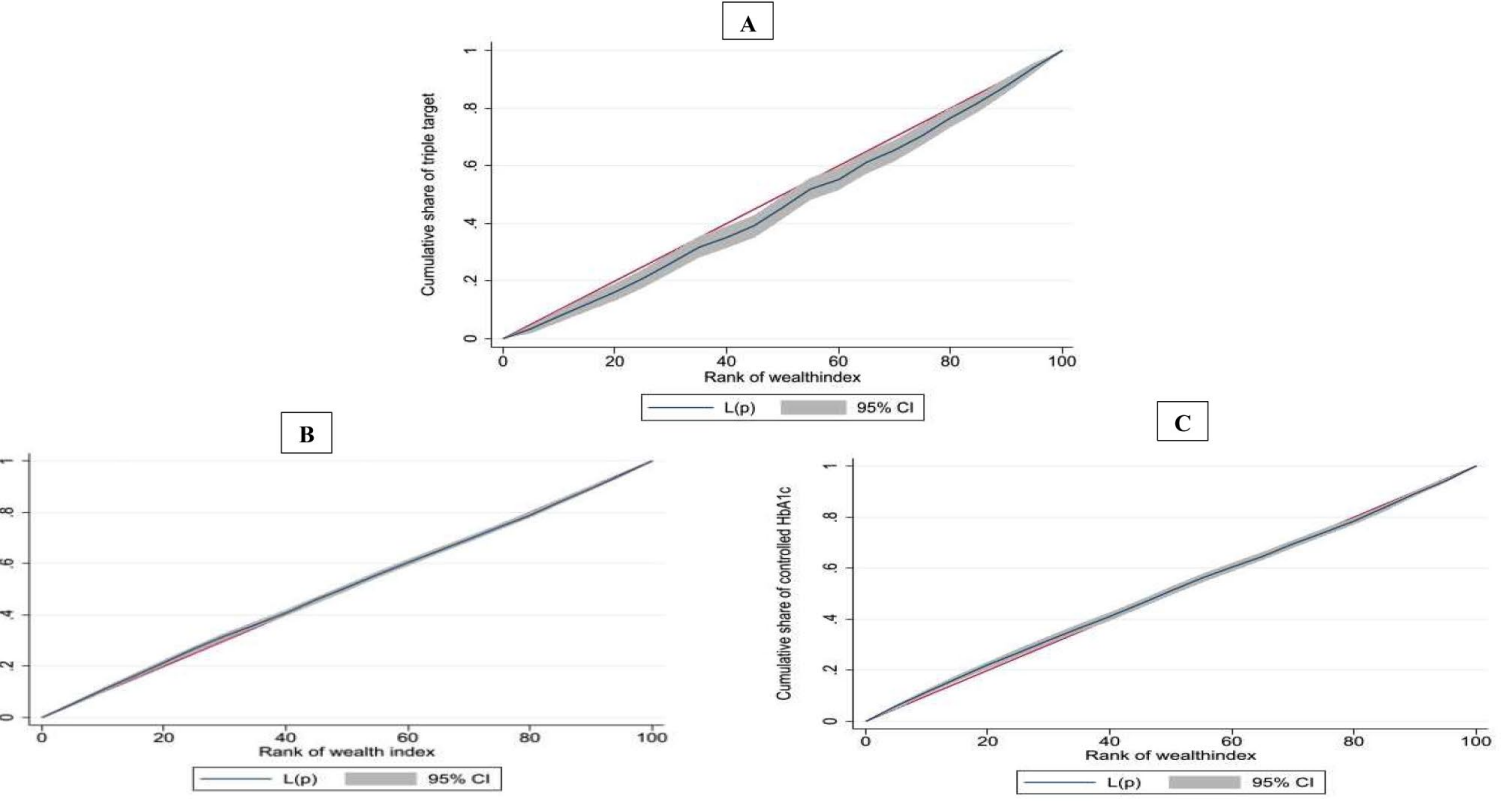
Lorenz curves (concentration curves) for **A**; controlled triple target, **B**; controlled FBG, **C**; controlled HbA1c

Figure 2 illustrates the concentration curves for T2DM variables. As shown, the concentration curve for the controlled triple target variable was below the equity diagonal line. The concentration curves fall below the line of equity, indicating a higher concentration among people with a higher SES.

In females, the CI for the controlled triple target was positive (0.209) and highly significant (*p* < 0.001). Similarly, this index for controlled FBG was positive (0.0367) and strongly significant (0.0001). Additionally, the CI for the controlled HbA1c in females was also positive (0.0745) and highly significant (*p* < 0.001) indicating a concentration of this variable among individuals with higher SES.

Furthermore, the findings revealed that the CI for the controlled triple target in 35–45 and 65–75 age groups was significantly positive (0.376 and 0.124, respectively), suggesting a favorable condition for this variable in individuals with higher SES.

In the 65–75 age group, the CI for the controlled FBG was positive (0.0708) and highly significant (*p* < 0.001) revealed that controlled FBG concentration was observed among people with higher SES. Similarly, the CI for controlled HbA1c in the 45–55 age group was positive (0.0607) and highly significant (*p* < 0.001).

In summary, the concentration of controlled T2DM variables, including the controlled triple target, controlled FBG, and controlled HbA1c, appears to be notably higher among individuals with higher SES across various demographic groups.

Another finding reveals that the CI for the controlled triple target among urban dwellers was positive (0.0967) and highly significant (*p* < 0.001). However, the CI for the controlled FBG among rural dwellers was negative (-0.044) and significant (0.002), indicating a higher concentration of the controlled FBG in people with lower SES. Additionally, the CI for controlled HbA1c in rural dwellers was negative (-0.0576, *p* = 0.0015), while in urban dwellers, it was positive (0.0306, *p* = 0.0009).

The CI for the controlled HbA1c in regions with medium prevalence of diabetes was positive (0.0534) and significant (0.0002), indicating that controlled HbA1c was concentrated in people with higher SES in these regions. The CI for the controlled FBG in regions with low prevalence was negative (-0.0277) and significant (0.0316), revealing that the concentration of controlled FBG variable was observed among people with lower SES. Additionally, the CI for the controlled Triple target in regions with high prevalence was significantly positive (0.124). In other words, in these regions, the controlled triple target was concentrated in people with higher SES.

**Table 3:** Concentration indices for CI for T2DM variables based on different demographic categories

Variables	Controlled triple target				Controlled FBG				Controlled HbA1c			
	CI	SE	P-value	p-value (Comparison)	CI	SE	P-value	p-value (Comparison)	CI	SE	P-value	p-value (Comparison)
**Total**	0.1067	0.0198	< 0.001	-	0.0125	0.0071	0.078	-	0.0324	0.0083	0.0001	-
**Sex**												
Women	0.209	0.0278	< 0.001	< 0.001	0.0367	0.0095	0.0001	0.0035	0.0745	0.0114	< 0.001	< 0.001
Men	-0.0002	0.028	0.993		-0.0048	0.0106	0.648		-0.0111	0.0122	0.363	
**Age group (years)**												
35–45	0.376	0.0467	< 0.001	0.006	-0.009	0.019	0.641	0.222	0.0223	0.0188	0.236	0.260
46–55	0.0357	0.0301	0.236		0.0116	0.0121	0.339		0.0607	0.0142	< 0.001	
56–65	0.045	0.043	0.299		0.0019	0.0125	0.873		0.0022	0.0162	0.890	
66–75	0.124	0.049	0.012		0.0708	0.0158	< 0.001		0.0360	0.0194	0.064	
**Place of Residence**												
Rural	0.0029	0.044	0.947	0.059	-0.044	0.0142	0.002	0.001	-0.0576	0.0172	0.0009	< 0.001
Urban	0.0967	0.023	< 0.001		0.009	0.008	0.276		0.0306	0.0096	0.0015	
**Region**												
Low prevalence	0.0681	0.0369	0.065	0.425	-0.0277	0.0127	0.0316	0.001	0.0086	0.0155	0.577	0.514
Medium prevalence	0.0586	0.0331	0.077		0.0009	0.0123	0.940		0.0534	0.0141	0.0002	
High prevalence	0.124	0.0347	0.0003		0.0186	0.0124	0.132		0.0262	0.0146	0.0727	

CI: concentration index, FBG; fasting blood glucose

The multivariate logistic regression analysis presented in Table 4 indicates a gradient relationship between the quintile of the asset index and the achievement of diabetes control parameters. Our analysis highlights a significant relationship between SES and diabetes control across various models and outcomes. For the Triple target control, a significant positive association was observed in the crude model for individuals in the richest quintile compared to the poorest (OR = 1.48, 95% CI = 1.14–1.92), suggesting that higher socioeconomic status substantially increases the likelihood of achieving diabetes management goals.

In the case of FBG, individuals in the second, third, and fourth quintiles demonstrated improved odds compared to the poorest quintile, with the fourth quintile showing a significant advantage in the crude model (OR = 0.81, 95% CI = 0.72–0.92) and maintained significance across Model 1 (OR = 0.85, 95% CI = 0.75–0.96) and Model 2 (OR = 0.82, 95% CI = 0.72–0.93), indicating a consistent benefit of higher SES on FBG control.

For controlled HbA1c, a significant improvement was also seen moving from the poorest to higher quintiles. Notably, the fourth quintile’s odds of achieving target HbA1c levels were significantly better across all models, peaking in Model 2 with an OR of 0.73 (95% CI = 0.63–0.84), highlighting the strong influence of socioeconomic status on glycemic control.

These findings illuminate the significant role of socioeconomic status, as measured by the asset index, in the achievement of diabetes control targets. The gradients observed suggest that interventions aiming at improving diabetes outcomes may need to consider socioeconomic factors as key components in their design and implementation strategies.

**Table 4:** Association quintile of the asset index status with diabetes control

	Controlled triple target		
Quintile of the asset index	Crude	Model 1	Model 2
Poorest	Ref.	Ref.	Ref.
2	1.19(0.91–1.57)	1.15(0.88–1.52)	1.11(0.84–1.46)
3	1.27(0.97–1.66)	1.21(0.92–1.59)	1.13(0.86–1.49)
4	**1.33(1.02–1.74)**	1.27(0.97–1.66)	1.13(0.86–1.50)
Richest	**1.48(1.14–1.92)**	**1.39(1.06–1.82)**	1.14(0.85–1.54)
	**Controlled FBG**		
	**Crude**	**Model** **1**	**Model 2**
Poorest	Ref.	Ref.	Ref.
2	**0.86(0.77–0.97)**	**0.89(0.79–0.99)**	**0.87(0.78–0.98)**
3	**0.88(0.78–0.98)**	0.91(0.81–1.02)	**0.89(0.79–0.99)**
4	**0.81(0.72–0.92)**	**0.85(0.75–0.96)**	**0.82(0.72–0.93)**
Richest	0.97(0.86–1.09)	1.04(0.92–1.17)	0.97(0.85–1.11)
	**Controlled HbA1c**		
	**Crude**	**Model 1**	**Model 2**
Poorest	Ref.	Ref.	Ref.
2	**0.84(0.74–0.95)**	**0.86(0.75–0.98)**	**0.83(0.73–0.95)**
3	**0.85(0.75–0.97)**	**0.87(0.76–0.99)**	**0.83(0.72–0.95)**
4	**0.78(0.68–0.89)**	**0.79(0.69–0.91)**	**0.73(0.63–0.84)**
Richest	0.97(0.86–1.11)	1.01(0.88–1.15)	0.87(0.79–1.01)

Multivariate logistic regression analyses

Crude model: Quintile of the asset index

Model 1: Quintile of the asset index, age, sex

Model 2: Quintile of the asset index, age, sex, education

## Discussion

This study aimed to analyze the socioeconomic inequalities in controlled T2DM variables in Iran. Our results suggest that the controlled triple target and controlled HbA1c variables were positively associated with individuals with higher SES, aligning with the findings of previous studies [8, 15, 18–21]. This result indicates that individuals with lower SES are less likely to seek diabetes care. One possible reason for this is the barriers to accessing medical services, including the healthcare costs, transportation expenses, and a lack of access to a proper diet [2, 8, 19–21]. Additionally, low SES is associated with lower levels of physical activity and unhealthy behaviors such as smoking [21, 22], which are risk factors contributing to the increased prevalence of diabetes.

We found that there is no significant difference in the concentration of controlled T2DM variables in sex groups. This suggests that individuals with lower SES may seek diabetes care less frequently. The prevalence of T2DM in females with high SES was low, which is consistent with findings from previous studies [12, 23, 24]. However, contrasting findings from other studies have indicated a high prevalence of diabetes among females [11, 12, 15, 18]. One possible explanation for this trend is the differing perceptions of health, health behavior, and lifestyle between males and females [23]. Males in the lowest SES often resort to harmful lifestyle behaviors, such as smoking, alcohol consumption, inactivity, and poor diet, as coping mechanisms in adverse and stressful situations [12].

According to another finding, there is no significant difference in the concentration of controlled T2DM variables in different age groups. Totally, the prevalence of T2DM in individuals with different age groups in highest SES was low. Its reasons can be attributed to easy access to health care, healthy foods, sports facilities, education, job opportunities and lifestyle choices of the highest SES [8].

Additionally, we found that HbA1c and FBG variables were better controlled among rural dwellers with low SES, indicating a more targeted focus on diabetes care in this demographic. The observed difference in diet between urban and rural areas, along with factors such as lack of physical activities, consumption of processed foods, and increased urbanization, may contribute to the higher prevalence of T2DM in urban dwellers [1, 19, 25]. Furthermore, successful control of T2DM has been observed in rural areas, attributed to the effective implementation of Primary Healthcare (PHC) and the management of non-communicable diseases [11].

Another finding indicates that controlled FBG is more concentrated among individuals with lower SES in areas with low prevalence. This could be attributed to the decreased demand in regions with low prevalence, potentially leading to increased accessibility to medical services for individuals with lower SES. Consequently, individuals with lower SES may find it easier to utilize the available medical services provided by healthcare centers.

The clear gradient in diabetes control across socioeconomic quintiles, especially noted in the significant findings for the Triple target, FBG, and HbA1c levels, underscores a crucial insight: socioeconomic status plays a significant role in the management of diabetes.

The persistence of this gradient across multiple models, even after adjusting for age, sex, and education, highlights the complex interplay between socioeconomic factors and health, suggesting that barriers to diabetes control extend beyond individual health behaviors to encompass broader social and economic factors.

The richest quintile’s significantly higher odds of achieving the Triple target in the crude model reflect the broad advantages conferred by higher SES, including better access to care and resources necessary for effective diabetes management.

The consistent significance of higher quintiles, particularly the fourth, in improving FBG control across all models, emphasizes the necessity for targeted healthcare strategies that address SES disparities. This finding is critical for healthcare providers and policymakers, suggesting that interventions focusing on middle to lower SES groups could yield substantial improvements in diabetes outcomes.

Furthermore, the robust association between higher SES and better HbA1c control, especially significant in the higher quintiles across all models, highlights the impact of socioeconomic factors on glycemic control. This relationship suggests that beyond medical treatment, addressing social determinants of health is vital for effective diabetes management.

These findings collectively highlight the need for comprehensive strategies that consider socioeconomic disparities in diabetes care. Healthcare policies must focus on reducing these gaps through targeted interventions, such as improving access to diabetes education and healthcare services for lower SES groups and supporting programs that address the broader determinants of health. Addressing these disparities is crucial for achieving more equitable health outcomes and underscores the importance of integrating social and economic support into diabetes care strategies.

Despite important findings, this study has certain limitations that should be acknowledged. Psychological factors and variables such as occupation, family size, smoking, body mass index, and other factors were not taken into account, potentially contributing to inequalities related to SES and impacting the study result. Additionally, the inability to access information on individual’s income led to the classification based solely on assets. Combining income and assets could have resulted in a more accurate and precise classification.

To the best of our knowledge, this is the first study to investigate the relationship between socioeconomic inequalities and T2DM in the Iranian population. The study’s strength lies in its adequate sample size.

## Conclusions

This study demonstrated that diabetes care is more concentrated among individuals with higher SES while the poorer groups experience lower quality care for T2DM due to various limitations. Consequently, it is imperative to designed implement programs and interventions aimed at controlling and preventing this disease. Recommended actions may include periodic screening particularly in deprived areas, initiatives to promote physical activity and healthy diets, improvement of living conditions, and enhanced accessibility to healthcare services.

## Data Availability

The data cannot be shared openly, but if requested, it can be obtained by contacting the corresponding author.

## Abbreviations

DM: Diabetes Mellitus
T2DM: Type 2 diabetes mellitus
DiaCare: Diabetes Care
HbA1c: Hemoglobin A1C
FBG: Fasting Blood Glucose
CIs: Concentration Indices
SES: Socio Economic Status
PCA: Principle component analysis
NCD: Non-Communicable Disease
IDF: International Diabetes Federation
ADA: American Diabetes Association
PHC: Primary Healthcare
